# Comparative Study of Redox Status of MDCK Cells in Chicken Embryo Extract Versus Fetal Bovine Serum

**DOI:** 10.3390/ijms27062794

**Published:** 2026-03-19

**Authors:** Jun-Hyun Kim, Jin-Mi Park, Mi-Kyung Nam, Seung-Min Hong, Eun-Ju Kim, Sun-Young Hwang, Kyoung-Ok No, Mee-Hyun Lee, Kang-Seuk Choi, Hyuk-Joon Kwon

**Affiliations:** 1 Laboratory of Avian Diseases, Department of Farm Animal Medicine, College of Veterinary Medicine and BK21 PLUS for Veterinary Science, Seoul National University, Seoul 08826, Republic of Korea; loupgarouhs@snu.ac.kr (J.-H.K.);; 2 Research Institute for Veterinary Science, College of Veterinary Medicine, Seoul 08826, Republic of Korea; 3 GeNiner Inc., Seoul 08826, Republic of Korea; 4 College of Korean Medicine, Dongshin University, Naju 58245, Republic of Korea; 5 Laboratory of Poultry Medicine, Department of Farm Animal Medicine, College of Veterinary Medicine and BK21 PLUS for Veterinary Science, Seoul National University, Seoul 08826, Republic of Korea; 6 Farm Animal Clinical Training and Research Center (FACTRC), GBST, Seoul National University, Pyeongchang 25354, Republic of Korea

**Keywords:** Madin–Darby canine kidney cells, fetal bovine serum, chicken embryo extract, serum alternative, redox state

## Abstract

Fetal bovine serum (FBS) is the standard supplement for cell culture, yet we previously demonstrated that it drives hyper-proliferation and phenotypic drift in Madin–Darby canine kidney (MDCK) cells, compromising their epithelial identity and ciliogenesis. In contrast, a modified chicken embryo extract (CEE) preserved these intrinsic properties, maintaining a stable and physiologically relevant phenotype. To elucidate the metabolic mechanisms driving these distinct cellular fates, we performed a comparative analysis of redox status and metabolomic profiles. We found that FBS forces a metabolic shift toward oxidative phosphorylation, resulting in mitochondrial stress characterized by elevated mitochondrial reactive oxygen species (mtROS), calcium overload, and the accumulation of uremic toxins like hippuric acid. Conversely, CEE established a balanced redox environment. Although CEE induced higher intracellular signaling ROS via NADPH oxidase 1/2, it prevented oxidative damage by upregulating antioxidant transcription factors, such as nuclear factor erythroid 2-related factor 2, and enzymes such as Mn superoxide dismutase. Additionally, metabolomic analysis revealed that CEE is enriched with antioxidants (ascorbic acid, proline) and signaling molecules (5-hydroxyindole-3-acetic acid). These findings indicate that while FBS imposes a metabolic burden leading to cellular stress, CEE provides a favorable metabolic microenvironment that supports homeostasis and epithelial integrity, validating its superiority as a culture supplement.

## 1. Introduction

Fetal bovine serum (FBS), which comprises a complex mixture of proteins, hormones, growth factors, and other bioactive molecules that facilitate cell proliferation and survival, is extensively utilized as a supplement for cell culture media [1]. However, the use of FBS is associated with various issues, including batch-to-batch variability, ethical concerns, and a relatively high cost [2]. These limitations have driven the search for alternative supplements capable of providing consistent and reliable support for cell growth and function [3,4].

Recently, we reported that a modified chicken embryo extract (CEE), which exhibits higher productivity than conventional CEE, can replace fetal bovine serum (FBS) as a supplement to maintain cell growth, viability, and intrinsic cellular properties by providing a broad range of nutrients, adhesion molecules, and growth factors essential for cell proliferation [5]. Understanding the potential benefits of CEE supplementation in cell culture systems is crucial. By addressing the limitations associated with FBS, CEE has the potential to enhance experimental consistency, mitigate ethical concerns, and improve the reliability of cell-based studies.

Previously, our next-generation sequencing (NGS) analysis revealed distinct transcriptomic landscapes between two groups of MDCK cells cultured under different supplementation conditions. MDCK-FBS cells, defined as MDCK cells maintained in FBS-supplemented medium, exhibited significant enrichment of genes involved in “cell cycle” and “DNA replication,” suggesting a hyper-proliferative state associated with phenotypic drift [5]. In contrast, MDCK-CEE cells, defined as MDCK cells cultured with CEE supplementation, showed upregulation of gene sets related to “cilium assembly” and “intraciliary retrograde transport,” indicating preservation of functional epithelial identity and maturation. Given that cellular metabolism and redox signaling are key modulators of both proliferative and differentiation-associated transcriptional programs, we hypothesized that the distinct phenotypes observed in our previous study are closely associated with differences in oxidative status between MDCK-FBS and MDCK-CEE cells.

Reactive oxygen species (ROS) are involved in the regulation of signaling pathways governing cellular behaviors such as proliferation, differentiation, and apoptosis [6,7,8,9]. Cellular ROS sources include leakage from the mitochondrial electron transport chain and from a number of ROS-generating plasma membrane and cytosolic enzymes [10]. During oxidative burst, large amounts of ROS are produced by plasma membrane NADPH oxidase (NOX), a well-characterized multicomponent enzyme [11]. When oxidative stress resulting from overproduction of ROS causes damage to cellular components, cells are defended by antioxidant systems such as nuclear factor erythroid 2-related factor 2 (NRF2), superoxide dismutase (SOD), catalase (CAT), and glutathione peroxidase (GPX) [12].

The redox state fosters a supportive environment for growth and development, as life depends on oxidation–reduction reactions, including mitochondrial processes crucial for energy extraction and maintaining cellular bioenergy and metabolism [13,14].

Furthermore, the metabolic environment provided by serum supplements can profoundly influence cellular redox homeostasis. While the kidney medulla, where MDCK cells originate, is characterized by a low-oxygen microenvironment favoring glycolysis [15,16], conventional FBS-based cultures often impose a metabolic shift toward oxidative phosphorylation, potentially leading to mitochondrial stress [5].

Although FBS has been reported to exhibit antioxidant properties by reducing malondialdehyde levels in rabbit spermatozoa [17] and enhancing antioxidant gene expression in porcine embryo cells [18], our findings demonstrate that in the specific context of MDCK renal epithelial cells, FBS exerts a significant oxidative burden and metabolic stress. This clear contrast strengthens the rationale for utilizing CEE as a superior alternative to mitigate such stress and maintain epithelial stability. Meanwhile, avian-derived supplements have demonstrated consistent protective effects; for instance, chick embryo egg hydrolysates possess significant antioxidant capacity, scavenging 2,2′-azino-bis(3-ethylbenzothiazoline-6-sulfonic acid) and 2,2-diphenyl-1-picrylhydrazyl radicals [19], while antioxidant-rich extracellular vesicles from chick embryo blood have been shown to alleviate oxidative stress and enhance cell survival in fibroblasts [20].

In this study, to investigate whether CEE creates a metabolically superior microenvironment that prevents oxidative stress-induced drift, we employed a multi-faceted approach integrating redox profiling and UPLC-QTOF-MS-based metabolomics. This metabolomic profiling was essential to bridge the gap between ROS dynamics and phenotypic stability, providing a comprehensive understanding of how metabolic shifts dictate cellular fate under different culture conditions.

## 2. Results

### 2.1. ROS and Ca^2+^ Levels in MDCK-CEE and MDCK-FBS Cells

**Figure 1:**
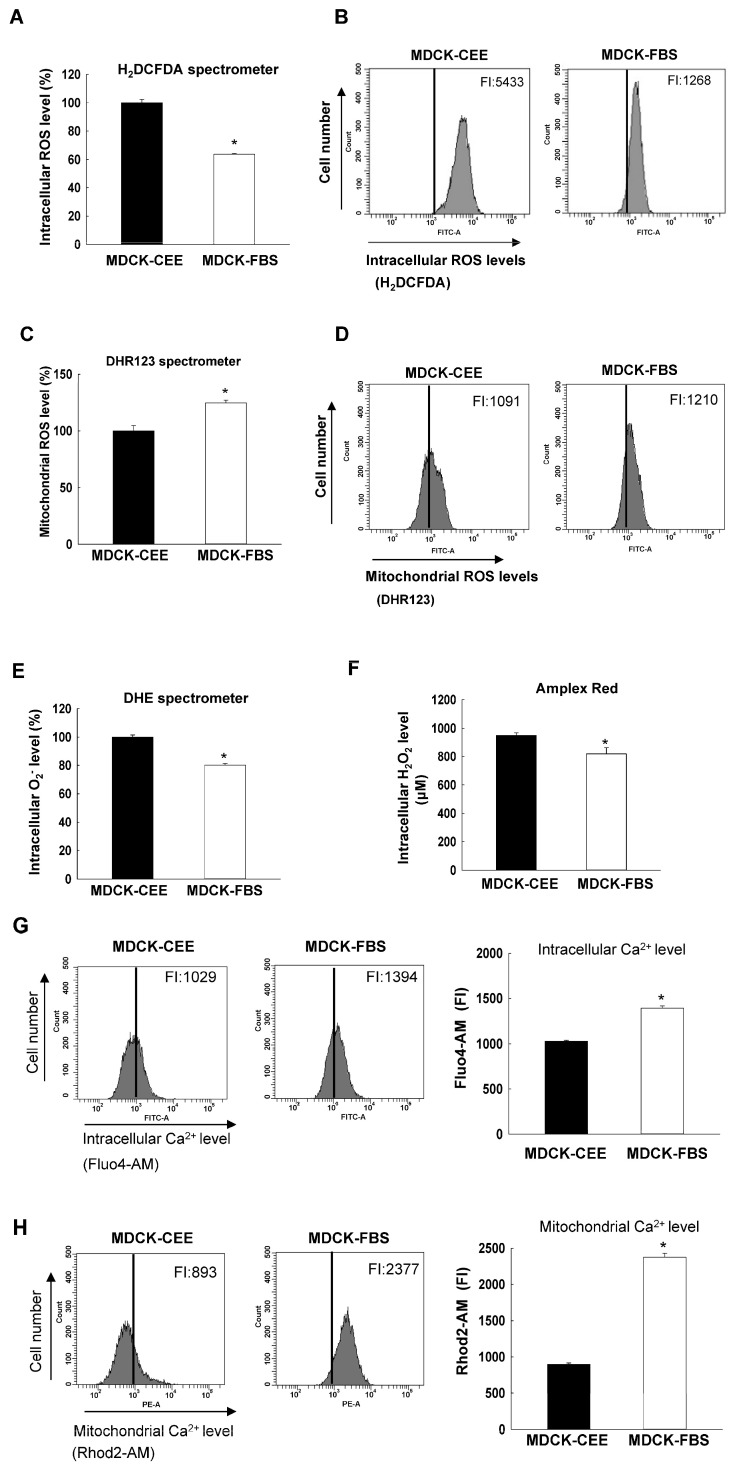
Redox status upon adapting MDCK cells to CEE and FBS. (**A**,**B**) Intracellular ROS levels of MDCK-CEE and MDCK-FBS cells were investigated through H_2_DCFDA staining using (**A**) a fluorescence spectrophotometer and (**B**) a flow cytometer. (**A**) * *p* < 0.05 compared with MDCK-CEE cells. Mitochondrial ROS levels were assessed using a fluorescence spectrophotometer (**C**) and a flow cytometer (**D**) after DHR123 staining. (**C**) * *p* < 0.05 compared with MDCK-CEE cells. (**E**) The level of superoxide anion radicals (O_2_**^·−^**) was detected using a fluorescence spectrophotometer after DHE staining. * *p* < 0.05 compared with MDCK-CEE cells. (**F**) The level of hydrogen peroxide (H_2_O_2_) was measured via an Amplex Red hydrogen peroxide assay using a fluorescence spectrophotometer. * *p* < 0.05 compared with MDCK-CEE cells. (**G**) The intracellular Ca^2+^ level was evaluated using a flow cytometer after Fluo4 AM staining. * *p* < 0.05 compared with MDCK-CEE cells. (**H**) The mitochondrial Ca^2+^ level was evaluated using a flow cytometer after Rhod2 AM staining. * *p* < 0.05 compared with MDCK-CEE cells. All error bars represent standard deviations from at least three independent experiments (n = 3). MDCK, Madin-Darby canine kidney; CEE, chicken embryo extract; FBS, fetal bovine serum; ROS, reactive oxygen species; H2DCFDA, 2′,7′-dichlorodihydrofluorescein diacetate; DHR123, dihydrorhodamine 123; DHE, dihydroethidium.

ROS generation in MDCK-CEE and MDCK-FBS cells was analyzed using various assays, and the results were compared. Overall, MDCK-CEE cells exhibited higher intracellular ROS levels but maintained lower mitochondrial ROS and Ca^2+^ levels compared to MDCK-FBS cells. Intracellular ROS levels, determined via 2′,7′-dichlorodihydrofluorescein diacetate (H_2_DCFDA) staining through fluorescence spectrophotometry and flow cytometry, were higher in MDCK-CEE cells than in MDCK-FBS cells, with fluorescence intensity (FI) values of 5433 for MDCK-CEE cells and 1268 for MDCK-FBS cells (Figure 1A,B). However, mitochondrial ROS levels, measured through dihydrorhodamine 123 (DHR123) staining, were higher in MDCK-FBS cells than in MDCK-CEE cells, with FI values of 1091 for MDCK-CEE cells and 1210 for MDCK-FBS cells (Figure 1C,D). Upon comparing the levels of superoxide anion radicals and hydrogen peroxide between the two cell types using dihydroethidium (DHE) and Amplex Red staining, both were higher in MDCK-CEE cells than in MDCK-FBS cells (Figure 1E,F). Intracellular Ca^2+^ levels, evaluated via Fluo4-acetoxymethyl (AM) staining, and mitochondrial Ca^2+^ levels, evaluated via Rhod2-AM staining, were higher in MDCK-FBS cells than in MDCK-CEE cells (Figure 1G,H).

### 2.2. Oxidative Cellular Damage Caused by ROS to MDCK-CEE and MDCK-FBS Cells

**Figure 2:**
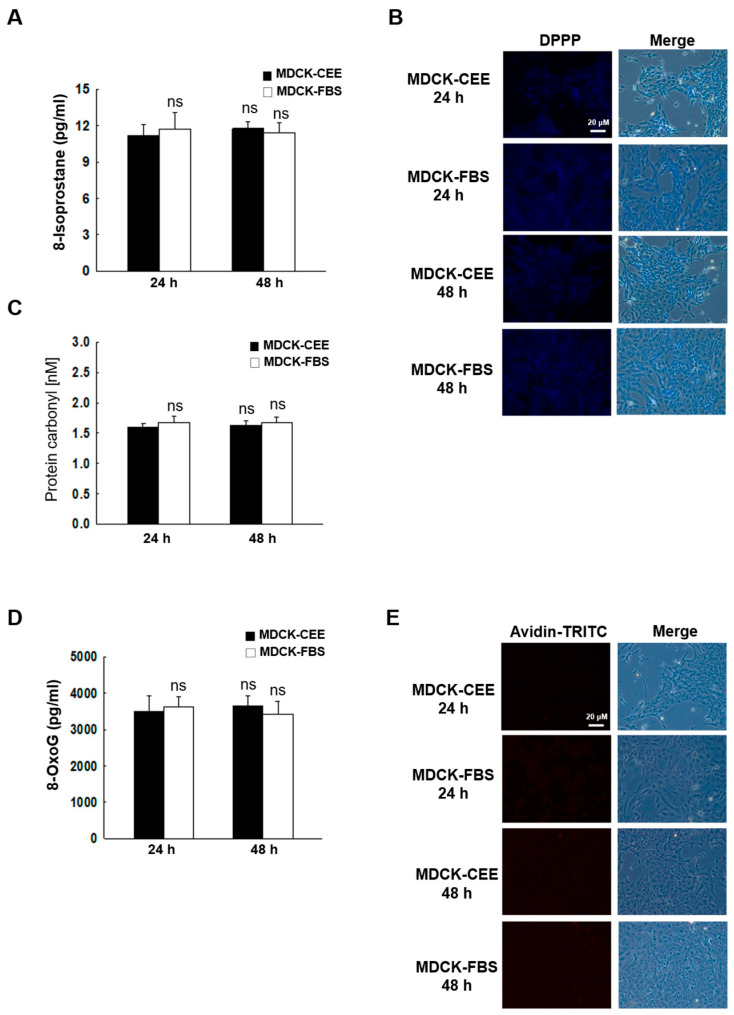
The comparison of damage to cellular macromolecules in MDCK-CEE and MDCK-FBS cells. (**A**) Lipid peroxidation was quantified by measuring 8-isoprostane levels. Statistical significance: ns, non-significant. Error bars represent standard deviations from at least three independent experiments (n = 3). (**B**) Lipid peroxidation was evaluated using confocal microscopy after DPPP staining. (**C**) Protein oxidation was measured by assessing carbonyl formation levels. Statistical significance: ns, non-significant. (**D**) DNA damage was evaluated by measuring 8-OxoG levels. Statistical significance: ns, non-significant. All error bars represent standard deviations from at least three independent experiments (n = 3). (**E**) Avidin-TRITC conjugate staining was employed to assess the formation of 8-OxoG, and images were obtained through confocal microscopy. DPPP, diphenyl-1-pyrenylphosphine; 8-OxoG, 8-oxoguanine.

We detected oxidative stress-induced lipid, protein, and DNA modifications in MDCK-CEE and MDCK-FBS cells using biomarkers of oxidative stress. No significant differences in lipid, protein or DNA oxidative damage markers were observed between MDCK-CEE and MDCK-FBS cells at 24 and 48 h (Figure 2A–E).

### 2.3. NOX Expression in MDCK-CEE and MDCK-FBS Cells

**Figure 3:**
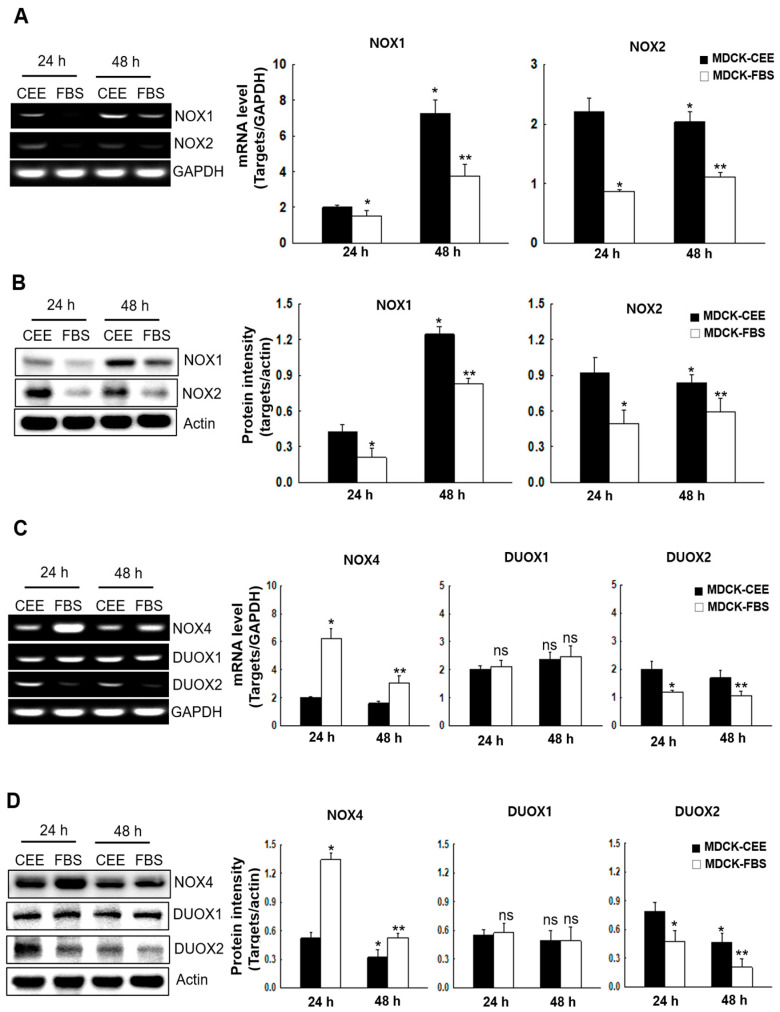
The expression of ROS-producing systems in MDCK-CEE and MDCK-FBS cells. (**A**,**B**) mRNA (**A**) and protein (**B**) expression of NOX1 and NOX2 was evaluated through qRT-PCR and a Western blotting assay and quantified. Statistical significance: * *p* < 0.05 vs. MDCK-CEE at 24 h; ** *p* < 0.05 vs. MDCK-CEE at 48 h; ns, non-significant. (**C**,**D**) mRNA (**C**) and protein (**D**) expression of NOX4, DUOX1, and DUOX2 was assessed through qRT-PCR and a Western blotting assay and quantified. Statistical significance: * *p* < 0.05 vs. MDCK-CEE at 24 h; ** *p* < 0.05 vs. MDCK-CEE at 48 h; ns, non-significant. All error bars represent standard deviations from at least three independent experiments (n = 3). GAPDH was used as the internal reference gene for normalizing the mRNA expression of all target genes. NOX, NADPH oxidase; qRT-PCR, quantitative real-time polymerase chain reaction; DUOX, dual oxidase; GAPDH, glyceraldehyde 3-phosphate dehydrogenase.

Because NOX1, NOX2, NOX4, dual oxidase 1 (DUOX1), and DUOX2 are predominantly expressed in MDCK cells, we compared their mRNA and protein expression levels in both cell types. To distinguish the effects of different reactive oxygen species, these enzymes were analyzed in two separate groups based on their primary products: superoxide anion radicals (NOX1 and NOX2) and hydrogen peroxide (NOX4, DUOX1, and DUOX2). Overall, MDCK-CEE cells showed an upregulated expression of superoxide-generating NOX enzymes, whereas hydrogen peroxide-generating enzymes showed mixed responses. The mRNA and protein levels of NOX1 and NOX2 were increased in MDCK-CEE cells (Figure 3A,B) [21]. In terms of the mRNA and protein levels of NOX4, DUOX1, and DUOX2, DUOX2 expression was increased in MDCK-CEE cells, and NOX4 expression was increased in MDCK-FBS cells [22]; however, DUOX1 expression showed no difference between the two cell types (Figure 3C,D).

### 2.4. Antioxidant Enzyme Expression in MDCK-CEE and MDCK-FBS Cells

**Figure 4:**
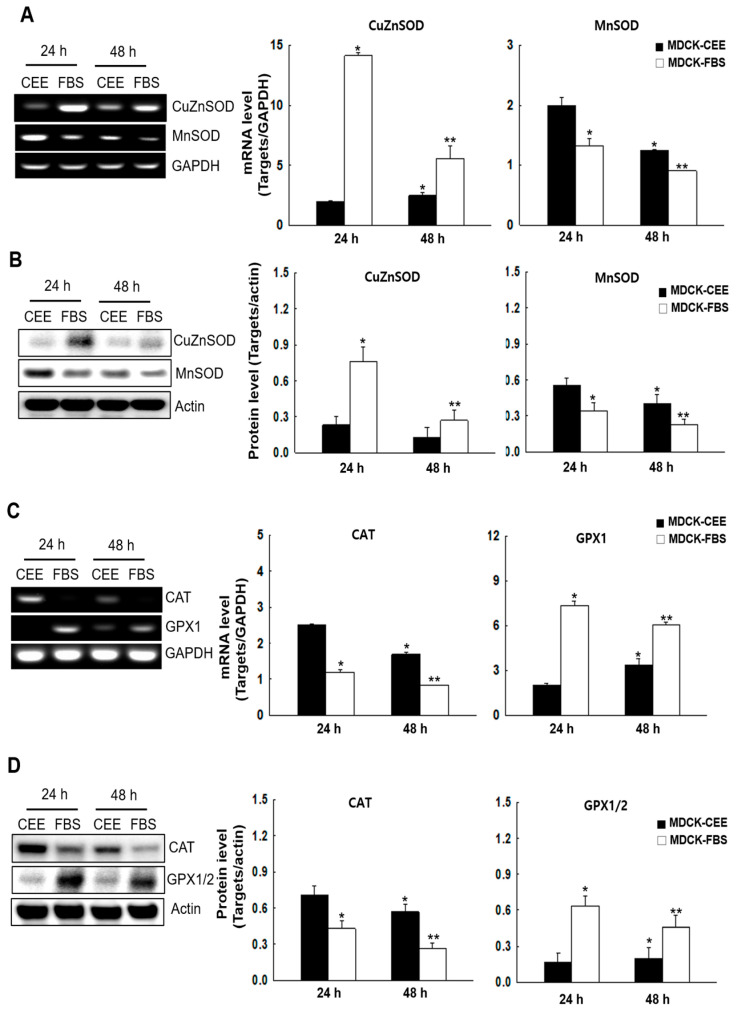

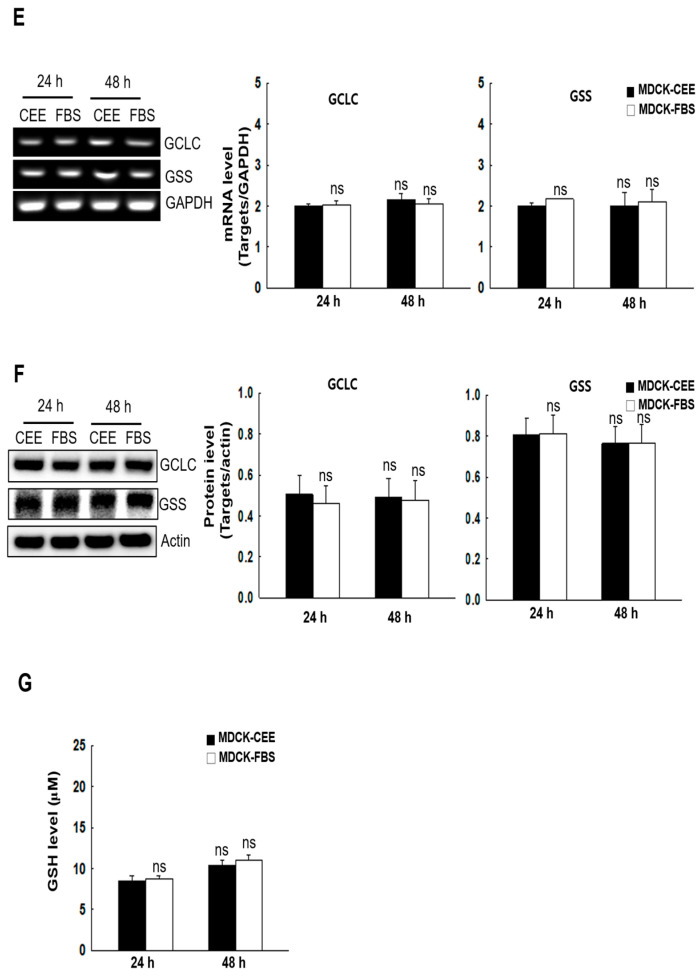
The expression levels of antioxidant-related systems in MDCK-CEE and MDCK-FBS cells. (**A**,**B**) mRNA (**A**) and protein (**B**) expression of CuZnSOD and MnSOD was evaluated through qRT-PCR and a Western blotting assay and quantified. Statistical significance: * *p* < 0.05 vs. MDCK-CEE at 24 h; ** *p* < 0.05 vs. MDCK-CEE at 48 h. (**C**,**D**) mRNA (**C**) and protein (**D**) expression of CAT and GPX1/2 was evaluated through qRT-PCR and a Western blotting assay and quantified. Statistical significance: * *p* < 0.05 vs. MDCK-CEE at 24 h; ** *p* < 0.05 vs. MDCK-CEE at 48 h; ns, non-significant. (**E**,**F**) mRNA (**E**) and protein (**F**) expression of GCLC and GSS was assessed through qRT-PCR and a Western blotting assay and quantified. (**G**) The GSH level was measured using a GSH detection kit. (**E**–**G**) Statistical significance: ns, non-significant. All error bars represent standard deviations from at least three independent experiments (n = 3). GAPDH was used as the internal reference gene for normalizing the mRNA expression of all target genes. SOD, superoxide dismutase; CAT, catalase; GPX, glutathione peroxidase; GCLC, glutamate-cysteine ligase catalytic subunit; GSS, glutathione synthetase; GSH, reduced glutathione.

After confirming the absence of spontaneous oxidative stress-induced cell damage in both MDCK-CEE and MDCK-FBS cells, we compared the mRNA and protein expression of their respective antioxidant systems. In general, the two cell types exhibited distinct antioxidant defense strategies, with MDCK-CEE cells primarily upregulating MnSOD and CAT, while MDCK-FBS cells favored CuZnSOD and GPX1/2. Regarding the enzymes that regulate O_2_^−^ and H_2_O_2_ levels [23], the expression of CuZnSOD was higher in MDCK-FBS cells than in MDCK-CEE cells, while MnSOD expression was higher in MDCK-CEE cells (Figure 4A,B). Similarly, CAT expression was higher in MDCK-CEE cells than in MDCK-FBS cells, whereas GPX1/2 expression was higher in MDCK-FBS cells (Figure 4C,D). Regarding the glutathione synthesis components [24], no significant differences were observed in the expression of glutamate-cysteine ligase catalytic subunit (GCLC), glutathione synthetase (GSS), or total reduced glutathione (GSH) between the two cell types (Figure 4E–G).

### 2.5. NRF2 Expression and Activity in MDCK-CEE and MDCK-FBS Cells

**Figure 5:**
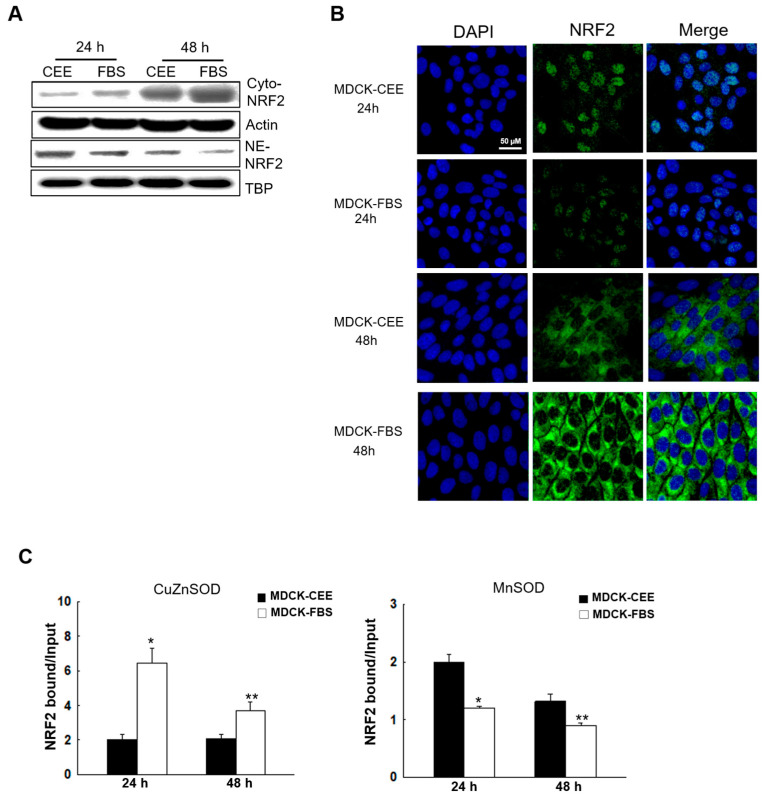
NRF2 activity in MDCK-CEE and MDCK-FBS cells. (**A**) Protein expression of NRF2 was assessed by conducting a Western blotting assay. Actin and TATA-box binding protein (TBP) were used as cytosolic and nuclear loading controls. (**B**) Nuclear translocation of NRF2 protein in MDCK-CEE and MDCK-FBS cells was photographed using confocal microscopy. Cell nucleus (DAPI: blue), NRF2 (green); scale bar = 50 μm. (**C**) A ChIP-qPCR assay was conducted at 24 h and 48 h in MDCK-CEE and MDCK-FBS cells using NRF2 antibodies. The enrichment of *CuZnSOD* and *MnSOD* promoter sequences in the DNA immunoprecipitated by NRF2 antibodies was assessed through qPCR and normalized to the input DNA. Statistical significance: * *p* < 0.05 vs. MDCK-CEE at 24 h; ** *p* < 0.05 vs. MDCK-CEE at 48 h. All error bars represent standard deviations from at least three independent experiments (n = 3). NRF2, nuclear factor erythroid 2-related factor 2; TBP, TATA-box binding protein; DAPI, 4′,6-diamidino-2-phenylindole; ChIP, chromatin immunoprecipitation.

Antioxidant-related genes such as SOD, CAT, and GPX have antioxidant response element (ARE) sequences in their promoter regions, and NRF2 is an important transcription factor that regulates the ARE-based expression of these genes [25]. In addition, NRF2 protein more often underwent nuclear translocation in MDCK-CEE cells compared to MDCK-FBS cells at 24 h, and this was maintained at 48 h (Figure 5A,B). In addition, there was an increase in NRF2 binding to the promoter region of CuZnSOD in MDCK-FBS cells, and to the promoter region of MnSOD in MDCK-CEE cells (Figure 5C). Consequently, the differential activation and promoter binding of NRF2 directly regulate the distinct expression patterns of downstream antioxidant enzymes, including CuZnSOD, MnSOD, CAT, and GPX, thereby shaping the overall cellular redox status.

### 2.6. Metabolomic Profiling of MDCK-CEE and MDCK-FBS Cells

**Figure 6:**
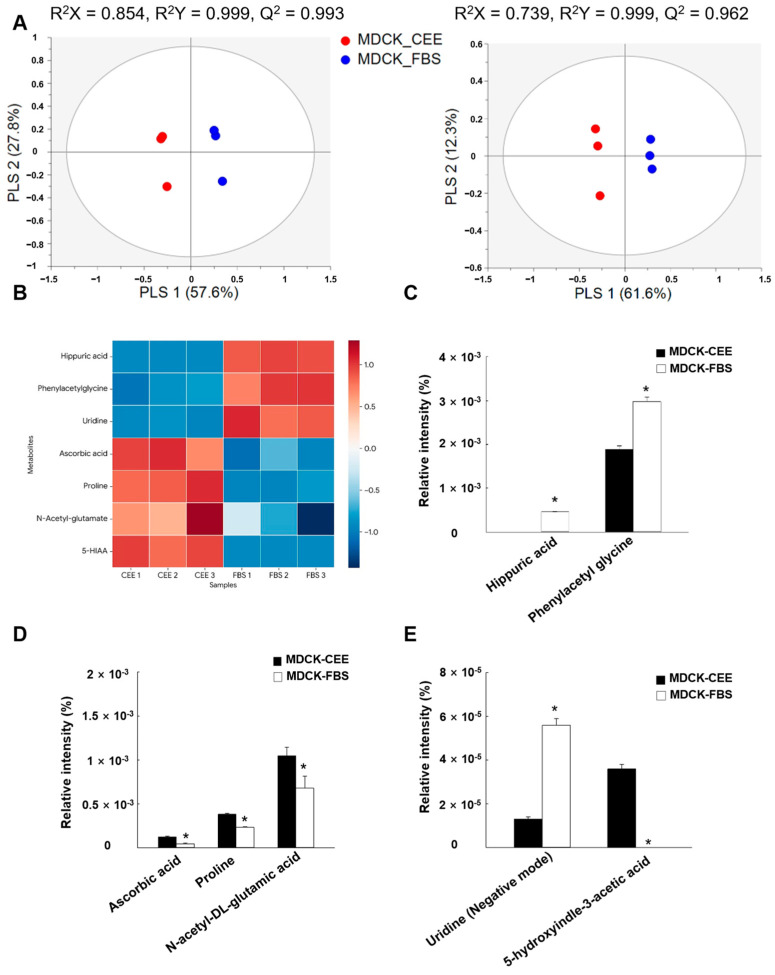
Metabolomic profiling and comparative analysis of metabolite abundance in MDCK-CEE and MDCK-FBS cells. (**A**) PLS-DA score plots of MDCK cells cultured in CEE (red) and FBS (blue) derived from UPLC-QTOF-MS data in positive (R^2^X = 0.854, Q^2^ = 0.993) and negative ion mode (R^2^X = 0.739, Q^2^ = 0.962). (**B**) Heatmap visualization of the top differentially regulated metabolites based on Z-score-normalized data. (**C**) Relative intensity (%) of the uremic toxins hippuric acid and phenylacetylglycine. (**D**) Relative intensity (%) of the antioxidants and glutathione precursors ascorbic acid, proline, and N-acetyl-DL-glutamic acid. (**E**) Relative intensity (%) of uridine and 5-hydroxyindole-3-acetic acid (5-HIAA). * *p* < 0.05; ns, not significant. All error bars represent standard deviations from at least three independent experiments (n = 3). PLS-DA, partial least squares discriminant analysis; UPLC-QTOF-MS, ultra-performance liquid chromatography-quadrupole time-of-flight mass spectrometry; 5-HIAA, 5-hydroxyindole-3-acetic acid.

To investigate the metabolic alterations associated with different serum supplements, we performed UPLC-QTOF-MS-based metabolomic profiling. Score plots from the partial least squares discriminant analysis (PLS-DA) show distinct separation between the two groups in both positive and negative ion modes (Figure 6A), and the heatmap of the top differentially regulated metabolites further illustrates these distinct metabolic signatures (Figure 6B).

Specific metabolites related to oxidative stress and antioxidant defense were analyzed. The uremic toxins hippuric acid and phenylacetylglycine were significantly elevated in MDCK-FBS cells compared to MDCK-CEE cells (Figure 6C), while MDCK-CEE cells exhibited significantly higher levels of antioxidants and glutathione precursors, including ascorbic acid, proline, and N-acetyl-DL-glutamic acid (Figure 6D). Additionally, uridine levels were significantly higher in MDCK-FBS cells, whereas 5-hydroxyindole-3-acetic acid (5-HIAA) was exclusively detected in MDCK-CEE cells (Figure 6E).

## 3. Discussion

Based on our previous observation that MDCK-FBS cells grow faster than MDCK-CEE cells [5], we hypothesized that differences in cellular redox status might underlie this growth discrepancy. This study aimed to test this hypothesis by comparing ROS production, antioxidant responses, and metabolic profiles between these two culture conditions.

ROS play important roles in normal cellular events such as proliferation, differentiation, and cellular physiological and metabolic functions, but excessive ROS induce oxidative stress and lead to processes that negatively affect cell growth [26,27,28]. Our data show that intracellular ROS levels are significantly higher in MDCK-CEE cells, which may be attributed to the elevated expression of NOX1, NOX2, and DUOX2. Interestingly, these increased ROS levels did not result in significant oxidative damage to lipids, proteins, or DNA. This absence of cytotoxicity aligns with the concept of ‘oxidative eustress,’ where physiological levels of ROS serve as essential signaling messengers rather than damaging agents [29]. Conversely, MDCK-FBS cells display higher mitochondrial ROS (mtROS) and mitochondrial Ca^2+^ levels. The mitochondrial Ca^2+^ accumulation suggests a sustained influx driven by the calcium-rich FBS environment, leading to mitochondrial calcium overload [30]. This, in conjunction with elevated mtROS, constitutes a chronic metabolic burden that predisposes mitochondria to permeability transition pore (mPTP) opening and forces the cells into a hypermetabolic state to maintain survival.

To counter ROS, cells rely on antioxidant systems regulated by the transcription factor NRF2. Our data suggest a functional causal link in the NOX-NRF2 axis. In MDCK-CEE cells, CEE-induced ROS function as an upstream signal that enhances the nuclear translocation of NRF2, which binds more efficiently to the *MnSOD* promoter, enhancing the expression of this critical mitochondrial antioxidant enzyme and catalase. In contrast, NRF2 in MDCK-FBS cells shows stronger binding to the *CuZnSOD* promoter, upregulating CuZnSOD and GPX. This synchronized activation and distinct promoter binding reflect the different antioxidant strategies employed by these cells to maintain redox homeostasis.

Our metabolomic analysis provides a biochemical rationale for these distinct redox profiles and their link to cellular fate. MDCK cells intrinsically rely on glycolysis rather than oxidative phosphorylation (OXPHOS) for energy production [5]. However, FBS culture forces a metabolic shift toward OXPHOS. This “metabolic mismatch” explains the elevated mtROS levels and is exacerbated by the accumulation of uremic toxins (hippuric acid and phenylacetylglycine) exclusively in MDCK-FBS cells, which induce mitochondrial dysfunction [31,32]. This FBS-driven OXPHOS state, alongside high uridine levels, fuels a hyper-proliferative state and activates the TGF-β/SMAD pathway, driving epithelial–mesenchymal transition (EMT) [33]. Conversely, the robust antioxidant capacity of MDCK-CEE cells is supported by the enrichment of ascorbic acid, proline [34,35], and N-acetyl-DL-glutamic acid, which enhances de novo glutathione synthesis. Furthermore, the unique presence of 5-HIAA [36], potentially alongside other intrinsic bioactive components of CEE [5], likely contributes to the modulation of redox signaling. By restoring the native glycolytic profile, MDCK-CEE cells minimize mitochondrial stress and maintain intrinsic epithelial integrity. Thus, CEE provides a protective metabolic microenvironment that prevents oxidative stress-induced phenotypic drift.

In summary, the specific redox and metabolic profiles dictating cellular fate in our study hold profound functional significance. The oxidative stress and metabolic burden imposed by FBS drive hyper-proliferation and induce cellular stress [5,30], thereby compromising epithelial stability. Conversely, the oxidative eustress and balanced metabolome facilitated by CEE are functionally critical for maintaining proper cell differentiation, physiological growth rates, and morphological epithelial integrity [29,36,37]. These findings underscore that maintaining intrinsic redox and metabolic homeostasis is essential for the preservation of epithelial identity in vitro [5,26,27,28].

CEE, like FBS, may exhibit batch-to-batch variability influenced by factors such as embryo developmental stage and extraction conditions. Although we sought to minimize such variability by strictly controlling the developmental window of the chicken embryos and standardizing the extraction protocol, residual biological heterogeneity cannot be entirely excluded. Therefore, while our findings reflect the characteristics of the standardized CEE preparation used in this study, validation using independently produced batches will be necessary to confirm reproducibility and broader applicability.

In addition, this study was conducted exclusively in MDCK cells, an immortalized epithelial cell line of canine origin. Thus, the observed differences between FBS- and CEE-supplemented conditions may be influenced by cell-line-specific metabolic and redox characteristics. Although CEE supplementation promoted epithelial stability and redox homeostasis in MDCK cells, these findings should not be generalized to other cell types without further investigation. Future studies using diverse primary and stem cell models will be required to determine whether the observed effects represent a broader biological principle or are context-dependent.

These findings highlight the complexity of redox regulation and underscore the potential of CEE as an alternative to FBS. CEE not only supports cell growth, but also enhances redox signaling pathways and antioxidant responses without inducing oxidative stress. This suggests that CEE could be a viable, ethical, and cost-effective alternative to FBS, with potential implications for cell culture methodologies and biotechnological applications.

In conclusion, while previous studies have highlighted the phenotypic benefits of serum alternatives, this study reveals the novel underlying mechanisms by comparing the distinct redox and metabolic signatures of MDCK cells cultured in CEE versus FBS. A key novelty of our findings is the identification of a distinct NOX-NRF2 axis response; CEE promotes ‘oxidative eustress’—utilizing signaling ROS to fortify intrinsic antioxidant defenses—whereas FBS induces harmful ‘oxidative stress’ and a metabolic mismatch [5,31]. Furthermore, our metabolomic profiling identifies 5-HIAA as a unique modulator of redox signaling exclusively present in CEE [36], which, together with the complex bioactive microenvironment of CEE [5], helps preserve epithelial identity. By elucidating these novel metabolic signatures and redox mechanisms, this study not only demonstrates why CEE is a superior alternative but also provides a deeper understanding of the microenvironmental factors necessary for maintaining physiologically accurate in vitro models [19,20].

## 4. Materials and Methods

### 4.1. Reagents and Antibodies

FBS and CEE were obtained from Gibco BRL (Grand Island, NY, USA; Cat. No. 16000044, Lot No.2719152P) and GeNiner Inc. (Seoul, Republic of Korea), respectively. The modified CEE used in this study was prepared from end-term embryos under a standardized protocol (Korean Patent No. KR101250542B1) to ensure biological consistency, as described in [5].

H_2_DCFDA, DHR123, DHE, Fluo4-AM, and Rhod2-AM were purchased from Thermo Fisher Scientific (Waltham, MA, USA). CAT, GPX1/2, GSS, NRF2, and actin were obtained from Santa Cruz Biotechnology Inc. (Santa Cruz, CA, USA); NOX1 was obtained from Novus Biologicals (Cambridge, UK); CuZnSOD (SOD1), MnSOD (SOD2), GCLC, NOX2, NOX4, DUOX1, DUOX2, phospho-NRF2, and TBP were obtained from Abcam (Cambridge, MA, USA); and goat anti-rabbit IgG (H + L) HRP and goat anti-mouse IgG (H + L) HRP secondary antibodies were obtained from Thermo Fisher Scientific.

### 4.2. Cell Culture

Madin–Darby canine kidney (MDCK; Korean Cell Line Bank, Seoul, Republic of Korea; [KCLB 10034]) cells (passage 10 to 18) were cultured in Dulbecco’s modified Eagle’s medium (DMEM, Life Technologies, Grand Island, NY, USA) supplemented with 10% FBS (MDCK-FBS cells) and 10% CEE (MDCK-CEE cells), and 1% penicillin-streptomycin (Gibco BRL, Grand Island, NY, USA) at 37 °C with 5% CO_2_. Although Eagle’s Minimum Essential Medium (EMEM) is often used as a standard maintenance medium, DMEM was chosen in this study to provide a nutrient-rich environment suitable for evaluating metabolic shifts as its higher concentrations of amino acids and vitamins better support the high metabolic demands required for these analyses. All cell lines were screened and confirmed to be negative for *Mycoplasma* contamination using a mycoplasma detection kit (InvivoGen, San Diego, CA, USA).

### 4.3. Detection of ROS Levels

Formation of intracellular and mitochondrial ROS was measured using H_2_DCFDA and DHR123, as described in [38]. MDCK-CEE and MDCK-FBS cells were seeded in 6-well plates at 0.8 × 10^5^ cells/well and incubated for 24 and 48 h. Cells were pre-loaded with 5 μM H_2_DCFDA or DHR123 in the culture medium for 30 min. Measurements were carried out using a fluorescence spectrophotometer (LS-5B; Perkin Elmer Inc., Waltham, MA, USA) or a flow cytometer (FACS LSRII) with the FACSDiva™ 6.0 software (Becton Dickinson, Mountain View, CA, USA). A total of 10,000 cells per sample were analyzed by flow cytometry, and all experiments were performed in triplicate. Moreover, to detect intracellular O_2_**^·−^**, cells were pre-loaded with 10 μM DHE in the culture medium for 30 min, and the fluorescent level of DHE was measured using a fluorescence spectrophotometer (LS-5B; Perkin Elmer Inc.). For H_2_O_2_ detection, cells were seeded, and after 24 and 48 h, the staining solution (50 μM of Amplex^®^ red reagent (Thermo Fisher Scientific, Waltham, MA, USA) and 0.1 U/mL of horseradish peroxidase in phosphate buffer) was added to each well to a final volume of 100 μL; the samples were then incubated for 30 min in the dark. Fluorescence was monitored at excitation/emission values of 485 nm/580 nm using a fluorescence spectrophotometer (LS-5B; Perkin Elmer Inc.).

### 4.4. Detection of Ca^2+^ Level

The cells were plated in 6-well plates at 0.8 × 10^5^ cells per well. After incubation for 24 and 48 h, 10 μM Fluo-4 AM or Rhod-2 AM was added to each well and incubated for 30 min at 5% CO_2_ and 37 °C. The dye loading solution was removed and washed with PBS buffer. The stained cells were assessed using flow cytometry (FACS LSRII) with the FACSDiva™ 6.0 software (Becton Dickinson).

### 4.5. Lipid Peroxidation Assay

Lipid peroxidation was analyzed through DPPP (Sigma-Aldrich, St. Louis, MO, USA) staining and by detecting 8-isoprostane levels. Cells were stained with DPPP for 30 min in the dark and analyzed using a confocal microscope (LSM 5 PASCAL; Carl Zeiss, Jena, Germany) equipped with a 20x objective lens [39]. The image files were processed using the ZEN black edition v2.3 software (Carl Zeiss, Jena, Germany). A commercial enzyme immune assay kit (8-Isoprostane ELISA Kit; Cayman Chemical Co., Ann Arbor, MI, USA) was used to detect 8-isoprostane, a lipid peroxidation marker, according to the manufacturer’s directions [40].

### 4.6. Protein Carbonyl Formation

Cell pellets were washed with PBS prior to lysis with protein carbonyl lysis buffer. After the protein levels were quantified, the lysates were loaded into a protein carbonyl assay plate and incubated overnight at 4 °C. The amount of protein carbonyl formed was determined using the Oxiselect protein carbonyl ELISA kit (Cell Biolabs, San Diego, CA, USA) [41].

### 4.7. Analysis of 8-OxoG Level

The level of 8-hydroxy-2′-deoxyguanosine (8-OHdG), a nucleoside of 8-OxoG, in DNA was quantified using the Bioxytech 8-OHdG ELISA kit (Oxis International, Tampa, FL, USA), and the quantity of 8-OHdG was considered to be proportional to the level of 8-OxoG [42]. In addition, the quantity of 8-OxoG was determined via a fluorescence binding assay using avidin, which binds to 8-OxoG with high affinity. The cells were fixed and permeabilized with ice-cold methanol for 15 min and subsequently incubated with Avidin-TRITC (Sigma-Aldrich, St. Louis, MO, USA) for 1 h at 20 °C [43]. 8-OxoG was visualized using a confocal microscope (LSM 5 PASCAL; Carl Zeiss) equipped with a 20x objective lens. The image files were processed using the ZEN black edition software (Carl Zeiss).

### 4.8. Quantitative Real-Time Polymerase Chain Reaction (qRT-PCR)

Cellular RNA was extracted from cells using the PURY RNA Plus kit (GenDEPOT, Barker, TX, USA) according to the manufacturer’s instructions. To ensure high purity, an on-column DNase I treatment was performed to remove genomic DNA contamination. Subsequently, 1 μg of total RNA was reverse-transcribed into complementary DNA (cDNA) using the PrimeScript™ RT reagent kit (TaKaRa, Dalian, China) in a total volume of 20 μL, with thermal cycling conditions of 37 °C for 15 min and 85 °C for 5 sec. Next, 1 μL of cDNA was reacted with 5 μL of 2 × SYBR green mixture, 0.5 μL of forward primer (5 μM), 0.5 μL of reverse primer (5 μM), and 3 μL of double-distilled water. qRT-PCR was conducted using a Bio-Rad iQ5 real-time PCR detection system (Bio-Rad Laboratories, Hercules, CA, USA), and the conditions were as follows: pre-denaturation for 10 min at 95 °C; 40 cycles of 95 °C for 15 sec and at 60 °C for 1 min. Relative gene expression levels were normalized to GAPDH and calculated using the 2–ΔΔCt method. The primer pairs (Bioneer, Daejeon, Republic of Korea) are shown in Table 1 and Table 2.

### 4.9. Western Blot

Cellular protein (30 μg) was subjected to electrophoresis on a 10% SDS-polyacrylamide gel, transferred to a nitrocellulose membrane, blocked with 3% bovine serum albumin, and then incubated with the corresponding primary antibodies (1:1000) overnight at 4 °C; following this, it was treated with the relevant secondary antibody (1:5000) at 20 °C for 1 h. Each corresponding protein band was measured using the ChemiDoc imaging system (Bio-Rad Laboratories, Bonney Lake, WA, USA) using an enhanced chemiluminescence Western blotting detection kit (Amersham, Hercules, CA, USA). Protein bands were analyzed using the ImageJ v1.54 software (NIH, Bethesda, MD, USA).

### 4.10. Detection of GSH Level

Intracellular GSH content was estimated using the Bioxytech GSH-400 enzymatic method (OXIS International, Portland, OR, USA) in accordance with the manufacturer’s instructions. A total of 0.8 × 10^5^ cell/mL of cellular protein was precipitated in 5% metaphosphoric acid, and the resulting supernatant was used for the test. The level of GSH was calculated according to standard curves of increasing GSH concentrations.

### 4.11. Immunohistochemistry

Cells were rinsed twice in ice-cold PBS, fixed with 4% formaldehyde in PBS (pH 7.4) for 10 min at 20 °C, washed, and incubated for 1 h in washing solution [0.25% Triton X-100 in PBS (PBST)]. They were then blocked with 1% BSA in PBST for 30 min at 20 °C. Next, they were incubated with NRF2 antibody in blocking solution overnight at 4 °C. After washing, sections were incubated for 1 h at 20 °C with Alexa Fluor 594 donkey anti-rabbit (Abcam, Cambridge, MA, USA) in blocking solution. Finally, cells were rinsed, drained, and fixed using Fluoroshield mounting medium with DAPI (for nuclear staining dye; Abcam, Cambridge, MA, USA), and then analyzed using a confocal microscope (LSM 5 PASCAL) equipped with a 20x objective lens. The image files were processed using the ZEN black edition software (Carl Zeiss). For immunofluorescence analysis, at least five random fields containing approximately 100 cells in total were analyzed per experimental group.

### 4.12. ChIP Assay

The ChIP assay was conducted using the SimpleChIP^®^ Enzymatic Chromatin IP Kit (Agarose Beads) (Cell Signaling Technology, Danvers, MA, USA) in accordance with the manufacturer’s instructions. Cells were cross-linked in 1% formaldehyde for 10 min at 20 °C, and the reaction was ended by adding glycine at a final concentration of 125 mM for 5 min. Cells were collected with 5 mL of PBS and centrifuged for 20 min at 5000 rpm, and pellets were resuspended in 1 mL of buffer A. The lysate was sonicated using a Qsonica Q700 sonicator (Qsonica, Newtown, CT, USA) to cleave it into fragments of 200–1000 bp. Each sonication cycle consisted of 10 sec at 23% amplitude followed by a 1 min rest period, which was repeated for a total of 15 cycles. Fragment size consistency was regularly verified by agarose gel electrophoresis prior to immunoprecipitation. Lysates were centrifuged for 30 min at 14,000 rpm, and the supernatants were transferred to a new tube and then quantified. One percent of the lysate was used to verify the amount of DNA for each immunoprecipitation. For each reaction, 400 μg of chromatin and 20 μL of ChIP-grade protein G agarose beads were used for immunoprecipitation with antibodies against NRF2, and normal rabbit IgG antibody was used as a negative control. Samples were immunoprecipitated overnight at 4 °C, and the beads were washed 5 times for 5 min with 1 mL of ice-cold immunoprecipitation buffer. After the final wash, immunoprecipitation buffer was removed, and immunoprecipitates were eluted with ChIP elution buffer. DNA that was purified from the immunoprecipitated complex was subjected to 40 cycles of PCR, and 2 μL of each ChIP-DNA was used to amplify the ARE promoter regions of SOD1 and SOD2 using the primers described in Table 3.

### 4.13. UPLC-QTOF-MS-Based Metabolomics Analysis

Metabolites were extracted from cell pellets using cold 80% methanol. The mixture was vortexed, incubated at −20 °C for 1 h, and centrifuged at 14,000 rpm for 10 min at 4 °C, and the supernatant was collected and filtered through a 0.22 μm PTFE filter before analysis. Chromatographic separation was performed using an ACQUITY UPLC system (Waters Corp., Milford, MA, USA) equipped with an ACQUITY UPLC HSS T3 column (2.1 mm × 100 mm, 1.8 μm; Waters Corp.). The column temperature was maintained at 40 °C, and the flow rate was set to 0.4 mL/min. The mobile phases consisted of 0.1% formic acid in water (Solvent A) and in acetonitrile (Solvent B).

Mass spectrometry was conducted using a Xevo G2-XS QTOF-MS (Waters Corp.) in both positive and negative electrospray ionization (ESI) modes. The capillary voltages were set at 3.0 kV (positive) and 2.5 kV (negative), with a source temperature of 120 °C and a desolvation temperature of 400 °C. Leucine enkephalin was used as the lock mass for real-time mass correction.

Raw data were acquired using MassLynx v4.2 software (Waters, Milford, MA, USA) and processed with Progenesis QI v3.0 software (Waters, Milford, MA, USA) for peak alignment, normalization, and peak picking. For each experimental group, three biological replicates were prepared, and each sample was analyzed in three technical replicates to ensure analytical reproducibility. Metabolites were identified based on accurate mass measurements with a mass error tolerance of ≤ 10 ppm. Identification was further supported by comparing precursor and product ion fragmentation patterns against reference databases, including the Human Metabolome Database (HMDB), METLIN, and MoNA. Compounds exhibiting mass similarity and isotope similarity of ≥ 90% were considered positively identified. Multivariate statistical analyses, including Principal Component Analysis (PCA) and PLS-DA, were performed using SIMCA v18.0 software (Umetrics, Sartorius Stedim Biotech AS, Umea, Sweden). Pathway analysis was conducted using MetaboAnalyst v5.0 software (McGill University, Montreal, QC, Canada).

### 4.14. Statistical Analysis

All experiments were performed in triplicate. Data are presented as mean ± standard deviation. Statistical analyses were conducted using SigmaStat v3.5 software (Systat Software Inc., San Jose, CA, USA). Prior to ANOVA, data normality was evaluated using the Shapiro–Wilk test. Comparisons between groups were evaluated using one-way analysis of variance (ANOVA) followed by Tukey’s post hoc test. Differences were considered statistically significant at *p* < 0.05.

## Figures and Tables

**Table 1:** Primer sequences of antioxidant-related genes.

Gene (Dog)	Primer (Dog)	Sequence (5′-3′)
*SOD1*	sense	TGGTGGTCCACGAGAAACGAGATG
	antisense	CAATGACACCACAAGCCAAACGACT
*SOD2*	sense	CGCCGCCTACGTGAACA
	antisense	CTCCAGCGCCTCCAGATACT
*CAT*	sense	TGAGCCCAGCCCTGACAAAATG
	antisense	CTCGAGCCCGGAAAGGACAGTT
*GPX1*	sense	GCAACCAGTTCGGGCATCAG
	antisense	CGTTCACCTCGCACTTCTCAAAA
*GCLC*	sense	CCAAGTCCCTCTTCTTTCCTG
	antisense	CGGAGACGGTGTATTCTTGTC
*GSS*	sense	CTGGAGCGGCTGAAGGACA
	antisense	AGCTCTGAGATGCACTGGACA
*GAPDH*	sense	TGTCCCCACCCCCAATGTATC
	antisense	CTCCGATGCCTGCTTCACTACCTT

**Table 2:** NOX family primer sequences.

Gene (Dog)	Primer (Dog)	Sequence (5′-3′)
*NOX1*	sense	CTGGGTAGTTAACCACTGGTTCTC
	antisense	GCTTTCTCATATGACAGGAAGGC
*NOX2*	sense	GCAATAACGCCACTAACCTGAG
	antisense	AGCAAGTCCGCAAACCACTC
*NOX4*	sense	GAAACTTCTGTTTGATGAAATAGC
	antisense	GTGAAGAGTCTTAGAAATTGAATTGG
*DUOX1*	sense	TGACCCACCACCTCTACATCC
	antisense	GATTAGTGCCGGGACCAGG
*DUOX2*	sense	ACGGCTTCCTCTCCAAGGAT
	antisense	CCTTGTCCTGGAAGCCTGAC
*GAPDH*	sense	TGTCCCCACCCCCAATGTATC
	antisense	CTCCGATGCCTGCTTCACTACCTT

**Table 3:** ChIP primer sequences.

Gene (Dog)	Primer (Dog)	Sequence (5′-3′)
*SOD1*	sense	CAAATGAGACGCTGTGGCCAAACT
	antisense	GGTTGCAGTACGCGAAATGGCA
*SOD2*	sense	GAGTATCTATAACCTGGTCCCAGCC
	antisense	GCTGAACCGTTTCCGTTGCTTCTTGC

## Data Availability

The data presented in this study are available in the Supplementary Materials (Tables S1 and S2).

## Supplementary Materials

The following supporting information can be downloaded at: https://www.mdpi.com/article/10.3390/ijms27062794/s1.

## Abbreviations

The following abbreviations are used in this manuscript:

FBS	Fetal bovine serum
CEE	Chicken embryo extract
MDCK	Madin–Darby canine kidney
ROS	Reactive oxygen species
MtROS	Mitochondrial reactive oxygen species
NOX	NADPH oxidase
NRF2	Nuclear factor erythroid 2-related factor 2
MnSOD	Manganese superoxide dismutase
5-HIAA	5-hydroxyindoleacetic acid
TIPS	Tech Incubator Program for Startups
PLS-DA	Partial least squares discriminant analysis
H_2_DCFDA	2′,7′-dichlorodihydrofluorescein diacetate
DHE	Dihydroethidium
GAPDH	Glyceraldehyde 3-phosphate dehydrogenase
